# Variants in *KCNJ11* and *BAD* do not predict response to ketogenic dietary therapies for epilepsy

**DOI:** 10.1016/j.eplepsyres.2015.10.003

**Published:** 2015-12

**Authors:** Natasha E. Schoeler, Costin Leu, Jon White, Vincent Plagnol, Sian Ellard, Mar Matarin, Gary Yellen, Elizabeth A. Thiele, Mark Mackay, Jacinta M. McMahon, Ingrid E. Scheffer, Josemir W. Sander, J. Helen Cross, Sanjay M. Sisodiya

**Affiliations:** a NIHR University College London Hospitals Biomedical Research Centre, Department of Clinical and Experimental Epilepsy, UCL Institute of Neurology, London, United Kingdom; b UCL Institute of Child Health, London, United Kingdom; c Department of Genetics Environment and Evolution, UCL Genetics Institute, London, United Kingdom; d Department of Statistical Genetics, University College London, London, United Kingdom; e Molecular Genetics, University of Exeter Medical School, Exeter, United Kingdom; f Department of Neurobiology, Harvard Medical School, Boston, MA, USA; g Department of Neurology, Massachusetts General Hospital, Boston, MA, USA; h Royal Children's Hospital, Melbourne, Australia; i Murdoch Children's Research Institute, Melbourne, Australia; j Epilepsy Research Centre, The University of Melbourne, Austin Health, Melbourne, Australia; k Departments of Medicine and Paediatrics, The University of Melbourne, Melbourne, Australia; l Florey Institute of Neurosciences and Mental Health, Austin Health, Melbourne, Australia; m Epilepsy Society, Chalfont St Peter, United Kingdom; n Stichting Epilepsie Instellingen Nederland (SEIN), Heemstede, The Netherlands; o Great Ormond Street Hospital for Children, London, United Kingdom; p Young Epilepsy, Lingfield, United Kingdom

**Keywords:** Genetic biomarker, Ketogenic diet, *KCNJ11*, *BAD*, Epilepsy, Seizures, KDT, ketogenic dietary therapies, SNP, single nucleotide polymorphism, MAF, minor allele frequency, CMH, Cochran–Mantel–Haenszel

## Abstract

In the absence of specific metabolic disorders, predictors of response to ketogenic dietary therapies (KDT) are unknown. We aimed to determine whether variants in established candidate genes *KCNJ11* and *BAD* influence response to KDT.

We sequenced *KCNJ11* and *BAD* in individuals without previously-known glucose transporter type 1 deficiency syndrome or other metabolic disorders, who received KDT for epilepsy. Hospital records were used to obtain demographic and clinical data. Two response phenotypes were used: ≥50% seizure reduction and seizure-freedom at 3-month follow-up. Case/control association tests were conducted with *KCNJ11* and *BAD* variants with minor allele frequency (MAF) > 0.01, using PLINK. Response to KDT in individuals with variants with MAF < 0.01 was evaluated.

303 Individuals had *KCNJ11* and 246 individuals had *BAD* sequencing data and diet response data. Six SNPs in *KCNJ11* and two in *BAD* had MAF > 0.01. Eight variants in *KCNJ11* and seven in *BAD* (of which three were previously-unreported) had MAF < 0.01.

No significant results were obtained from association analyses, with either KDT response phenotype. *P*-values were similar when accounting for ethnicity using a stratified Cochran–Mantel–Haenszel test.

There did not seem to be a consistent effect of rare variants on response to KDT, although the cohort size was too small to assess significance.

Common variants in *KCNJ11* and *BAD* do not predict response to KDT for epilepsy. We can exclude, with 80% power, association from variants with a MAF of >0.05 and effect size >3. A larger sample size is needed to detect associations from rare variants or those with smaller effect sizes.

## Highlights

• Common *KCNJ11* and *BAD* variants were not associated with KDT response.
*•* There was no consistent effect of rare variants on KDT response.
• Larger cohorts may show associations from variants with effect size <3 or MAF < 0.05.
• Variants with small effect sizes are unlikely to be clinically relevant.
• Variants in other genes may influence response to KDT.

## Introduction

Ketogenic dietary therapies (KDT) can be an effective treatment for people with drug-resistant epilepsy (Henderson et al., 2006, Keene, 2006, Neal et al., 2008, Payne et al., 2011) and are the treatment of choice for epilepsy associated with the genetic conditions glucose transporter type 1 deficiency syndrome and pyruvate dehydrogenase complex deficiency (Kossoff et al., 2009).

In the absence of specific metabolic disorders, predictors of response to KDT are unknown (Schoeler et al., 2013). Certain epilepsies, many of which are caused by single gene mutations, may respond well to KDT (Kossoff et al., 2009, Nangia et al., 2012, Thammongkol et al., 2012), although further evidence is needed that epilepsies of different aetiologies respond differently to KDT. Strain-specific responsiveness to KDT in terms of seizure threshold has been shown in mice (Dutton and Escayg, 2008). From animal models, two genes have been highlighted as candidates implicated in the antiepileptic mechanisms of KDT (Giménez-Cassina et al., 2012): *KCNJ11*, which encodes the Kir6.2 pore-forming subunit of K_ATP_ channels, and *BAD*, which encodes the minimal death domain BH3-only protein and also has a metabolic role (Danial et al., 2003, Danial et al., 2008).

*Bad*^*S155A*^ (from mice bearing a non-phosphorylatable knock-in allele of *BAD*) and *Bad*^*−/−*^ cortical neurons and astrocytes exhibited increased mitochondrial utilisation of β-hydroxybutyrate and decreased utilisation of glucose, compared with wild types (Giménez-Cassina et al., 2012). These changes in energy substrate utilisation are reminiscent of the response to fasting or KDT. *Bad*^*S155A*^ and *Bad*^*−/−*^ mice showed resistance to kainic acid-induced seizures; resistance was diminished in *Bad*^*−/−*^, *Kir6.2*^*−/−*^ mice. The open probability of single K_ATP_ channels was increased in *Bad*-deficient neurons, compared to wild types.

Gene expression changes can directly influence phenotypic traits; a major influence on gene expression is genetic variation (Fu et al., 2012, Grundberg et al., 2010, Williams et al., 2007). It therefore seems possible that individual genetic variation influences the efficacy of KDT on seizure control. We hypothesised that variants in *KCNJ11* and *BAD* may influence response to KDT in humans.

## Materials and methods

### Ethics and recruitment

The project gained ethical approval through relevant ethics committees or institutional review boards. Written informed consent was obtained from all study participants or from their parents in the case of minors or adults with intellectual disability.

Participants were recruited from April 2011–December 2012 from the following sites: Great Ormond Street Hospital for Children, London; National Hospital for Neurology and Neurosurgery, London; Evelina Children's Hospital, London; St George's Hospital, London; Young Epilepsy (including Matthew's Friends clinics for Ketogenic Dietary Therapies), Surrey; Birmingham Children's Hospital, Birmingham; Addenbrooke's Hospital, Cambridge; Alder Hey Children's Hospital, Liverpool; Bristol Royal Hospital for Sick Children, Bristol, all in the UK; The Royal Children's Hospital, Melbourne, Australia; Austin Health, Melbourne, Australia. *KCNJ11* sequencing data were received from patients from Massachusetts General Hospital, in collaboration with Harvard Medical School, Boston, USA.

Criteria for study inclusion were: individuals aged ≥3 months who were either following KDT or who had followed KDT in the past for epilepsy. Exclusion criteria were: individuals who discontinued KDT before the 3-month point due to lack of tolerability (those who discontinued KDT before the 3-month point due to lack of response or seizure increase were included), individuals with known glucose transporter type 1 deficiency syndrome, pyruvate dehydrogenase complex deficiency or known to have other metabolic disorders, and individuals with progressive myoclonic epilepsies or other progressive neurological diseases.

In the UK clinics, all eligible for recruitment were invited to participate. The majority of Australian participants were recruited prospectively.

### Phenotypic data collection

All underwent electroclinical phenotyping to establish seizure type and epilepsy syndrome. This involved medical history, seizure semiology, examination and review of EEG and imaging studies. Demographic data were obtained from medical records. The proforma used to collect phenotypic information is given in Supplementary Material. Apart from KDT response, detailed clinical data were not available for cases from Boston.

KDT response was defined in terms of seizure frequency. In 121 cases, response was determined prospectively and in 125 cases it was retrospective Response was estimated in 28 day epochs prior to starting the diet (baseline) and prior to 3-month follow-up after the start of KDT. Clinic letters and seizure diaries, where already used as part of clinical monitoring (in 35 [11.6%] patients), were used to estimate seizure frequency at each time point. The calculation used to determine percentage reduction in seizure frequency was: [(*a* − *b*)/*a*] × 100, where *a* = number of seizures in the 28 days prior to KDT initiation; *b* = number of seizures in the 28 days preceding the 3-month point (three months since KDT was started). A case/control study design was adopted. Those with ≥50% seizure reduction were classified as ‘responders’; those with <50% seizure reduction were ‘non-responders’. Seizure freedom achieved at any follow-up point was also documented. All cases received from Boston had diet response classified at the 3-month point.

For individuals with demographic data available, the effect of various factors on KDT response was assessed by logistic regression. Statistics were performed in R (R: A Language and Environment for Statistical Computing, Vienna, Austria). To avoid the inflated likelihood of Type I error when testing multiple hypotheses, a Bonferroni-corrected significance threshold was applied.

### Genotypic data collection

DNA was extracted from venous blood using Autogen (AutoGen Inc, Hollister, Massachusetts, USA), Fujifilm (FUJIFILM Corporation, Tokyo, Japan) or QiaAmp Blood Maxi kits (Qiagen GmbH, Hilden, Germany).

*KCNJ11* and *BAD* Sanger sequencing was undertaken in a clinically-accredited laboratory at the Royal Devon & Exeter Hospital. Exon 1 of *KCNJ11* was amplified in three fragments, and the three exons in *BAD* were amplified using M13 tailed PCR primers (primers on request). Sequencing was performed using a Big Dye Terminator Cycler Sequencing Kit (Applied Biosystems, Warrington, UK) according to manufacturer's instructions. Reactions were analysed on an ABI 3130 Capillary sequencer (Applied Biosystems, Warrington, UK) and sequences were compared to the published sequence (*KCNJ11*: NM_000525; *BAD*: NM_004322.3) using Mutation Surveyor v.4.0.6 (SoftGenetics, Pennsylvania, US). Identified variants were checked for known mutations (Flanagan et al., 2009). Common single nucleotide polymorphisms (SNPs) were identified using public variant databases (dbSNP http://www.ncbi.nlm.nih.gov/SNP/, 1000 Genomes http://www.1000genomes.org/ and Exome Variant Server http://evs.gs.washington.edu/EVS/).

*KCNJ11* sequencing results were also received from Boston. The full *KCNJ11* gene had been sequenced bi-directionally using three 500–600 bp amplicons with published primers (Inoue et al., 1997). Sequencing was performed using an ABI 377 machine (PE Biosystems, NYSE:PEB) with Big Dye terminator chemistries (PE Biosystems, NYSE:PEB). Sequence traces were analysed using the GAP4 program of the Staden package (http://www.mrc-lmb.cam.ac.uk/pubseq).

### Power calculations

Power calculations were conducted using PGA Power Calculator (Menashe et al., 2008). An alpha level according to the effective number of tests was used (two phenotypes and seven SNPs were effectively tested for in association analyses—two SNPs were in high linkage disequilibrium). As there is no naturally-existing ketogenic diet (or indeed sustained calorie restriction, at least in Europeans), disease (or trait) prevalence is hard to gauge. The estimated prevalence of treatment-resistant epilepsy, the usual subject population considered for KDT, was therefore used: a disease prevalence of epilepsy of 0.5% was used, as in other studies (Kasperaviciute et al., 2010), of which ∼35% are thought to be treatment-resistant. The genetic architecture of response is unknown and so power calculations were performed using co-dominant, dominant and recessive penetrance models.

**Fig. 1:**
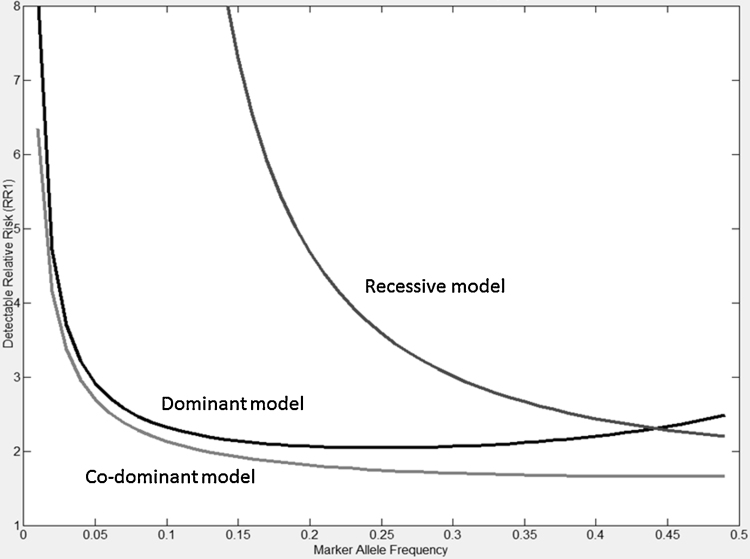
Detectable relative risk and disease allele frequency curves for a cohort of 303 people, with 80% power, assuming a disease prevalence of 0.00175, an alpha of 0.004, 126 cases and control:case ratio of 1.4 (assuming response as ≥ 50% seizure reduction).

As shown in Fig. 1, with a sample size of 303 (as used in the *KCNJ11* analysis), variants with a minor allele frequency (MAF) of approximately 0.05 and a relative risk of 3 could be detected with 80% power, assuming a dominant or co-dominant penetrance model; assuming a recessive penetrance model, the variant MAF would have to be at least 0.3 if the variant had a relative risk of approximately 3.

### Association analyses

The following quality control filtering was undertaken: individuals were excluded for missing genotype rates >2% and excessive identity-by-descent estimates indicating genotypic relatedness (Pi-hat [proportion of identity-by-descent] score >0.4, calculated from genome-wide SNP data, which were available for 243 individuals); SNPs with deviation from Hardy Weinberg equilibrium, *p*-value <1 × 10^−6^, or missing rates >2%, were excluded (cases from Boston were analysed separately, as these did not have *BAD* sequencing data).

Fisher's exact test for allelic association was conducted in PLINK (http://pngu.mgh.harvard.edu) (Purcell et al., 2007) for *KCNJ11* and *BAD* variants with MAF > 0.01. Two response phenotypes were examined: one, with response defined as ≥50% seizure reduction at 3-month follow-up and another, with response defined as seizure freedom at 3-month follow-up.

The Cochran–Mantel–Haenszel (CMH) test was also conducted to correct for population stratification. Each cluster was set as an ethnic group, which was self-reported by participants as genome-wide SNP data were not available for all individuals. The self-reported ethnicity clusters were: Caucasian (including Australian and American Caucasians, *n* = 251), African (*n* = 5), Middle Eastern (*n* = 6), Central/South Asian (*n* = 14), East Asian (*n* = 2), Black and Caucasian mix (*n* = 19), East Asian and Caucasian mix (*n* = 3), South Asian and Caucasian (*n* = 2) and South American (*n* = 1).

Unadjusted *p*-values were corrected using the permutation procedure (100,000 permutations).

Response to KDT in individuals with rare (MAF < 0.01) variants in *KCNJ11* and *BAD* was evaluated.

The predicted functional impact of variants with MAF > 0.01 in this cohort was obtained from various algorithms using wANNOVAR (http://wannovar.usc.edu) (Chang and Wang, 2012): SIFT (Sorting Intolerant From Tolerant) (Ng and Henikoff, 2003), PolyPhen2 (Polymorphism Phenotyping v2) (Adzhubei et al., 2010), LRT (likelihood ratio test) (Chun and Fay, 2009), Mutation Taster (Schwarz et al., 2010) and GERP++ (Genomic Evolutionary Rate Profiling) (Davydov et al., 2010). These scores were only available for non-synonymous variants. GERP++ scores for synonymous variants were obtained from National Heart, Lung, and Blood Institute (NHLBI) Exome Variant Server, NHLBI GO Exome Sequencing Project (ESP) (http://evs.gs.washington.edu/EVS). Variants were also looked up in Phenotype–Genotype Integrator (http://www.ncbi.nlm.nih.gov/gap/phegeni), The Human Gene Mutation Database (http://www.hgmd.cf.ac.uk) (Stenson et al., 2014) and NCBI (National Center for Biotechnology Information) Variation Viewer (www.ncbi.nlm.nih.gov/sites/varvu) to obtain information regarding human phenotypic-genotypic associations and functional class of the variants, and in dbSNP (http://www.ncbi.nlm.nih.gov/SNP/) to obtain MAF information.

The predicted functional impact of variants with MAF < 0.01 not present in dbSNP was determined from Alamut reports (v2.2, Interactive Biosoftware LLC, Rouen, France): function class, SIFT score, nucleotide and amino acid conservation, and Mutation Taster score. Variants not present in dbSNP were also sought in the Exome Aggregation Consortium Browser (ExAC, Cambridge, MA (URL: http://exac.broadinstitute.org) [December 2014 accessed]).

The predicted functional impact of variants with MAF < 0.01 present in dbSNP was obtained from various algorithms using wANNOVAR.

## Results

### Cohort demographics

Two individuals were excluded because 3-month KDT response data were not available; two further individuals were excluded due to lack of *KCNJ11* and *BAD* sequencing data. 246 individuals had *KCNJ11* and *BAD* sequencing data and KDT response data available. The demographic characteristics of these 246 participants are summarised in Table 1.

A further 57 individuals from Boston had *KCNJ11* sequencing results and KDT response data available (*KCNJ11* sequencing data were available for 58 individuals from Boston but response data were not available for one of these individuals). 176/303(58%) were responders at the 3-month point; 22/303(7%) were seizure-free. No demographic factor was significantly associated with KDT response at 3-month follow-up, for either response phenotype (see Supplementary Table 1). A Bonferroni-corrected significance threshold was set, based on an alpha of 0.05 and 28 tests (corrected threshold = 0.002; two phenotypes and 14 demographic factors).

### Association analyses

Six SNPs in *KCNJ11* and two in *BAD* had a MAF of >0.01 in our cohort and so were used in the association analyses; all these variants were previously-reported in dbSNP and ExAC. These variants are listed in Supplementary Table 2, with their predicted functional impact. Two variants in *KCNJ11*, rs5215 and rs5219, were in high LD (*r*^2^ = 0.9933 in our cohort, calculated using PLINK) and present with the same haplotype in 166 individuals.

No SNPs were removed due to deviation from Hardy Weinberg equilibrium or missingness rates. 243 individuals had genome-wide SNP data available; one pair of siblings was removed due to relatedness. *KCNJ11* and *BAD* sequencing failed in three subjects. 176 responders and 127 non-responders were included in the *KCNJ11* analysis; 126 responders and 120 non-responders were included in the *BAD* analysis.

None of the permutation-corrected p-values, from Fisher or CMH tests, with either KDT response phenotype, reached the formal threshold for a significant association (see Table 2, Table 3). rs5216 in *KCNJ11* had an uncorrected *p*-value (*p* = 0.019) that surpassed the threshold for suggestive significance (1/number of tests), in the ≥50% seizure reduction response phenotype analysis.

### Variants with MAF < 0.01

Eight variants in *KCNJ11* (all of which were previously reported in dbSNP or ExAC) and seven in *BAD* (of which three were previously reported in dbSNP or ExAC) had MAF < 0.01 in our cohort. KDT response in cases with these variants, with the predicted functional impact of these variants, is given in Supplementary Table 3.

Five variants have a predicted impact on protein structure. NM_00525.3:c.[451G > A];[=], p.Val151Met in *KCNJ11* encodes a highly-conserved amino acid (conserved up to Tetraodon [pufferfish]), has a deleterious SIFT classification and is classified as disease-causing by Mutation Taster. This variant was found in one non-responder. The missense variant rs41282930 in *KCNJ11* also has a predicted functional impact and was present in six individuals with a range of responses to KDT. NM_00525.3:c.[817A > G];[=] p.Ser273Gly in *KCNJ11* (present in one partial responder) and NM_004322.3:c. [226T > A];[=] p.Tyr76Asn in *BAD* (present in one responder and one non-responder) had deleterious SIFT classifications but were not predicted to be damaging by all other algorithms. NM_004322.3:c.[142G > A];[=] p.Ala48Thr in *BAD* (present in one non-responder) was not predicted to be damaging by either SIFT or Mutation Taster and was not highly conserved.

## Discussion

We evaluated the effect of variation in candidate genes *KCNJ11* and *BAD* on response to KDT. No significant results were obtained from our association studies, with either of our response phenotypes. With a sample size of 303, we had 80% power to detect associations of variants with a MAF of ≥0.05 and relative risk of ≥3, depending on the genetic model assumed. Variants in *KCNJ11* or *BAD* may have a smaller effect on KDT response, which we did not have power to detect. However, such effect sizes are unlikely to be clinically relevant when attempting to predict response to dietary therapies, though variants with smaller effect sizes may still cast light on the mechanisms of action of KDT.

rs5216 in *KCNJ11* merits possible further investigation as it reached suggestive significance in the ≥50% seizure reduction phenotype analysis. In pancreatic β cells, the binding of adenine nucleotides to the Kir6.2 pore closes the channel; variants affecting this subunit could influence the open-channel probability and alter cell excitability (Babenko et al., 2006). Considering the ‘K_ATP_-glycolysis hypothesis’ (Yellen, 2008), if rs5216 were to alter K_ATP_ open-channel probability in the brain, this may affect the extent to which KDT depress neuronal excitability. The functional consequences of rs5216 on K_ATP_ channel activity are unknown. It is located in a conserved region and is rare in the general population, indicating that it may influence gene/protein function and may be damaging (Liu and Kumar, 2013). rs5216, however, is a synonymous variant and is predicted by SIFT to be tolerated.

The previously-unreported variants and those SNPs with MAF < 0.01 in this cohort are unlikely to be important for most people following KDTs. They are not variants that influence response to KDT shared across patients; most variants were only present in one or two individuals. The possibility cannot be discarded that other, rare variants in *KCNJ11* or *BAD*, not found in this cohort, may play a minor role in response to KDT. Deep resequencing with application of stringent genotype call probabilities may be the only way to identify the full extent of rare genetic variation in an individual (Coventry et al., 2010).

This study has limitations. Due to the nature of dietary interventions such as KDT and the limited available of KDT in the UK, the sample size in this study is numerically small. Approximately 250 people were following the KD for epilepsy in the UK in 2011–2012 and 264 started the KD in 2012–2013 (personal communication, Katherine Lord, Head of Nutrition and Dietetics at Southmead Hospital and chairperson of Ketogenic Diet Professional Advisory Group, UK). Further international collaboration would be needed to obtain a larger cohort size. The time-points at which response is calculated also influence results. For many people with epilepsy, ‘responder’ or ‘non-responder’ status to KDT is known by the 3-month point, but it may take longer to categorise response for others, for example, those with seizure clusters. Alternative procedures to estimate response may need to be explored. *p*-Values were largely unaffected when including ethnicity as a covariate; whilst genetic influence on response to KDT might genuinely not be confounded by population stratification, it is difficult to be certain with our small number of non-European participants. In future studies, the issue of ethnicity and response to KDT should not be disregarded, as certain variants may be more relevant in populations with a more recent or longer history of survival through starvation. Most such populations, however, will not have epilepsy services with capacity for KDT, if indeed any treatment is available.

Our heterogeneous cohort is typical of individuals with complex epilepsy who commence KDT. Conducting association studies with narrower phenotypes, where patients are in more homogenous groups, for example, by epilepsy cause or syndrome, may be an option for future analyses.

## Conclusion

We have shown that common variants in the candidate genes *KCNJ11* and *BAD* are not associated with response to KDT at 3-month follow-up. Associations from variants with a smaller effect size or rare variants may be detected with a larger sample size. It remains to be seen whether variants in other genes influence response to KDT. An unbiased approach across the genome or exome is needed. This may allow us to identify biological pathways not previously associated with response to KDT (Minihane, 2013).

## Conflict of interest statement

This study was partly funded by the Wellcome Trust (084730). NES is supported by a UCL Impact Studentship in conjunction with Epilepsy Society. MM is supported by Epilepsy Research UK (F1206). JWS receives research support from Epilepsy Society, the Dr. Marvin Weil Epilepsy Research Fund, Eisai, GSK, WHO, EU FP7 and the National Institutes of Health (NIH), and has been consulted by and received fees for lectures from GSK, Eisai and UCB Pharma. JHC has received funds to the department for research into the ketogenic diet from Vitaflo. Honoraria for speaking have also been made to the department on her behalf from Nutricia. JHC and IS have written a cookery book ‘Ketocooking’, funds from the sale of which will be donated to their respective departments. SMS receives research support from Epilepsy Society, The Wellcome Trust, The European Commission, Dravet Syndrome UK, Epilepsy Action, MRC, NIH and The Katy Baggott Foundation and has received research support/fees from lectures from Eisai, GSK and UCB Pharma. The remaining authors have no conflicts of interest.

## Figures and Tables

**Table 1:** Cohort clinical data (for cases with diet response data, *n* = 246).

Gender	Male *n* = 129 (52%)
Female *n* = 117 (48%)
Age at seizure onset (years) *median (IQR)*	0.67 (0.2–2) (unknown for 1 case)
Age at diet onset (years) *median (IQR)*	5.70 (3.2–9.9)
Cause of epilepsy*	Genetic *n* = 30 (12.2%)
Structural-metabolic *n* = 70 (28.5%)
Unknown cause *n* = 146 (59.3%)
Epilepsy syndrome*	Dravet syndrome/severe myoclonic epilepsy of infancy *n* = 15 (6.1%); Lennox–Gastaut syndrome/LGS-spectrum *n* = 11 (4.5%); childhood absence epilepsy *n* = 3 (1.2%); juvenile myoclonic epilepsy *n* = 2 (0.8%); juvenile absence epilepsy *n* = 3 (1.2%); epilepsy with myoclonic atonic (astatic) seizures (Doose syndrome) *n* = 14 (5.7%); epilepsy with myoclonic absences *n* = 1 (0.4%); epilepsy with myoclonic atonic seizures and myoclonic absences *n* = 2 (0.8%); myoclonic epilepsy (unspecified) *n* = 7 (2.8%); epilepsy of infancy with migrating focal seizures *n* = 3 (1.2%); ohtahara syndrome *n* = 1 (0.4%); west syndrome *n* = 15 (6.1%); undiagnosed *n* = 169 (68.7%)
Number of AEDs at diet onset *mean [95% CI]*	2.33 [0.98–3.69]
Number of failed AEDs prior to diet onset *mean [95% CI]*	6.66 [4.84–8.49] (unknown for 3 cases)
Diet type (at 3-month point)**	Classical Ketogenic diet *n* = 161 (65.4%)
Medium Chain Triglyceride Ketogenic Diet *n* = 47 (19.1%)
Modified Atkins Diet *n* = 38 (15.4%)
Feed	Oral *n* = 167 (67.9%)
Gastrostomy/naso-gastric tube *n* = 63 (25.6%)
Oral and tube *n* = 16 (6.5%)

* Cause of epilepsy (*genetic*, *structural/metabolic*, *unknown*) and epilepsy syndromes have been classified according to Berg et al. (2010).

** No patients were following the Low Glycaemic Index Treatment, as this was not offered as a diet option at the study sites. If a patient transitioned to a different diet type before the 3-month point, the new/second diet type was considered this individual's ‘diet type’.

**Table 2:** Results of association analyses: common and intermediate variation in *KCNJ11* and *BAD* in responders (≥50% seizure reduction or seizure freedom at 3-month follow-up) and non-responders of KDT: *n* = 303 for *KCNJ11* and *n* = 246 for *BAD*.

With KDT response defined as ≥50% seizure reduction at 3-month follow-up								
Gene	SNP rs number	Location (build 37/hg19)	Minor allele (in ketogenic diet cohort)	Frequency of minor allele in non-responders	Frequency of minor allele in responders	Unadjusted *p*-value	Odds ratio [95%CI]	*p*-Value (100,000 permutations)
*KCNJ11*	rs8175351	11: 17,408,496	A	0.02756	0.02557	1	1.08 [0.3969–2.939]	1
rs1800467	11: 17,408,831	G	0.07087	0.04261	0.1481	1.714 [0.8466–3.468]	0.6355
rs5219	11: 17,409,572	A	0.3268	0.3381	0.7938	0.9504 [0.6745–1.339]	0.9997
rs5215	11: 17,408,630	A	0.3268	0.3381	0.7938	0.9504 [0.6745–1.339]	0.9997
rs5218	11: 17,409,069	T	0.248	0.304	0.1429	0.7552 [0.5247–1.087]	0.6122
rs5216	11: 17,408,838	G	0.007874	0.03977	0.01872	0.1916 [0.04316–0.8507]	0.132
*BAD*	rs34882006	11: 64,051,823	A	0.05	0.03175	0.3645	1.605 [0.6445–3.998]	0.9406
rs2286615	11: 64,039,175	T	0.1125	0.1706	0.07133	0.6161 [0.3671–1.034]	0.362

With KDT response defined as seizure freedom at 3-month follow-up								
*KCNJ11*	rs8175351	11: 17,408,496	A	0.02491	0.04545	0.326	0.5365 [0.118–2.439]	0.9043
rs1800467	11: 17,408,831	G	0.05872	0	0.1594	n/a	0.6116
rs5219	11: 17,409,572	A	0.3399	0.25	0.2489	1.544 [0.7636–3.124]	0.7493
rs5215	11: 17,408,630	A	0.3399	0.25	0.2489	1.544 [0.7636–3.124]	0.7493
rs5218	11: 17,409,069	T	0.2776	0.3182	0.6018	0.8234 [0.4253–1.594]	0.9856
rs5216	11: 17,408,838	G	0.02847	0	0.6207	n/a	0.9962
*BAD*	rs34882006	11: 64,051,823	A	0.04202	0	1	n/a	1
rs2286615	11: 64,039,175	T	0.145	0.0625	0.7126	2.543 [0.3306–19.56]	0.9986

**Table 3:** Results of association analyses: common and intermediate variation in *KCNJ11* and *BAD* in responders (≥50% seizure reduction or seizure freedom at 3-month follow-up) and non-responders of KDT, including ethnicity as a covariate: *n* = 303 for *KCNJ11* and *n* = 246 for *BAD*.

With KDT response defined as ≥50% seizure reduction at 3-month follow-up				
Gene	SNP rs number	Unadjusted *p*-value	Odds ratio [95%CI]	*p*-Value (100,000 permutations)
*KCNJ11*	rs8175351	0.9842	1.01 [0.369–2.766]	1
rs1800467	0.1668	1.656 [0.8072–3.396]	0.7343
rs5219 and rs5215	0.9602	1.009 [0.7099–1.434]	1
rs5218	0.1712	0.7728 [0.5342–1.118]	0.7514
rs5216	0.01847	0.1975 [0.0444–0.8784]	0.1318
*BAD*	rs34882006	0.3133	1.598 [0.6387–3.997]	0.9329
rs2286615	0.07559	0.6219 [0.3682–1.05]	0.4272

With KDT response defined as seizure freedom at 3-month follow-up				
*KCNJ11*	rs8175351	0.2264	0.3954 [0.08367–1.869]	0.8182
rs1800467	0.1063	n/a	0.5337
rs5219 and rs5215	0.07526	1.977 [0.9215–4.241]	0.377
rs5218	0.4768	0.7799 [0.3936–1.545]	0.9951
rs5216	0.2451	n/a	0.8889
*BAD*	rs34882006	0.4203	n/a	0.9835
rs2286615	0.4221	2.274 [0.2915–17.73]	0.9843
