# Supplementary material for: Variants in KCNJ11 and BAD do not predict response to ketogenic dietary therapies for epilepsy
Source: Epilepsy Res. 2015 Dec;118:22–8. doi: 10.1016/j.eplepsyres.2015.10.003 (PMC4819482; doi:10.1016/j.eplepsyres.2015.10.003)
Supplement: Supplementary file 1 [file mmc1.docx]

Supplementary Material

Phenotype proforma

| **Clinical history** |  | |
| --- | --- | --- |
| **Height**  **Weight** | **Pre-diet:** | **3 month point:** |
|  | **Pre-diet:** | **3 month point:** |
| **Epilepsy syndrome** |  | |
| **Seizure types** (before and during KD) |  | |
| **AEDs/other medications** | **Pre-diet:** | **3 month point:** |
|  | **Previous (start date, stop date, max. dose, response and side effects):** | |
| **Family history**  (epilepsy and other) |  | |
| **Neurodevelopment** |  | |
| **Neurological findings** |  | |
| **EEG data** |  | |
| **Imaging data**  CT  MRI  PET  SPECT |  | |
| **Date started KDT** |  | |
| **Date stopped KDT (if retrospective)** |  | |
| **Response to KDT** |  | |
| **Biochemical data** | **Pre-diet:** | **3 month point:** |
| **Side effects** |  | |
| **Long term outcome** | **6 month point:** | |
|  | **1 year:** | |
|  | **18 months:** | |
|  | **2 years:** | |
|  | **Post-2 years:** | |

Supplementary Table 1: Putative factors affecting response to KDT at 3-month follow-up

| **Factor** | **Number of responders with data available** | **Number of non-responders with data available** | **P values*** |
| --- | --- | --- | --- |
| **Gender** | 125 ≥50% seizure reduction | 121 <50% seizure reduction | 0.44 |
|  | 8 seizure-free | 238 not seizure-free | 0.14 |
| **Age of seizure onset (years)** | 125 ≥50% seizure reduction | 120 <50% seizure reduction | 0.84 |
|  | 8 seizure-free | 237 not seizure-free | 0.47 |
| **Age of diet onset (years)** | 125 ≥50% seizure reduction | 121 <50% seizure reduction | 0.29 |
|  | 8 seizure-free | 238 not seizure-free | 0.07 |
| **Aetiology** | 125 ≥50% seizure reduction | 121 <50% seizure reduction |  |
|  | 8 seizure-free | 238 not seizure-free |  |
|  | Genetic vs Structural/Metabolic | | 0.79 |
|  |  |  | 0.20 |
|  | Genetic vs Unknown | | 0.78 |
|  |  |  | 0.42 |
|  | Structural/Metabolic vs Unknown | | 0.44 |
|  |  |  | 0.42 |
| **Number of AEDs taken at diet onset** | 125 ≥50% seizure reduction | 121 <50% seizure reduction | 0.86 |
|  | 8 seizure-free | 238 not seizure-free | 0.62 |
| **Number of failed AEDs prior to diet onset** | 123 ≥50% seizure reduction | 120 <50% seizure reduction | 0.06 |
|  | 8 seizure-free | 235 not seizure-free | 0.79 |
| **Diet type** | 125 ≥50% seizure reduction | 120 <50% seizure reduction |  |
|  | 8 seizure-free | 237 not seizure-free |  |
|  | Classical Ketogenic diet vs Modified Atkins Diet | | 0.09 |
|  |  |  | 0.99 |
|  | Classical Ketogenic diet vs Medium Chain Triglyceride Ketogenic diet | | 0.41 |
|  |  |  | 0.85 |
|  | Modified Atkins Diet vs Medium Chain Triglyceride Ketogenic diet | | 0.44 |
|  |  |  | 0.99 |
| **Feed** | 125 ≥50% seizure reduction | 121 <50% seizure reduction |  |
|  | 8 seizure-free | 238 not seizure-free |  |
|  | Oral vs Tube | | 0.30 |
|  |  |  | 0.36 |
|  | Oral vs Oral and Tube | | 0.27 |
|  |  |  | 0.39 |
|  | Tube vs Oral and Tube | | 0.62 |
|  |  |  | 0.81 |

*p-values (Pr(>|z|)) obtained from *glm* function. Conducted once with response phenotype defined as ≥50% seizure reduction and once defined as seizure-freedom.

Supplementary Table 2: SNPs in *KCNJ11* and *BAD* with MAF>0.01 in our cohort, used in association analyses

| **Gene** | **SNP rs number** | **Location**  **(build 37/hg19)** | **Variant nomenclature**  **(cDNA level and protein level)*** | **Function class** | **Minor Allele Frequency**  **(1000 Genomes Project)** | **dbNSFP SIFT score and class** | **Polyphen2**  **score and class** | **LRT score and class** | **Conservation score GERP++** | **Mutation Taster score and class** |
| --- | --- | --- | --- | --- | --- | --- | --- | --- | --- | --- |
| ***KCNJ11*** | rs5219 | 11: 17,409,572 | NM_000525.3:c.67A>G  p.Glu23Lys | missense | 0.274 | 0.75  Tolerated | 0  Benign | 0.927728 Neutral | 3.65 | 0  Polymorphism |
|  | rs5218 | 11: 17,409,069 | NM_000525.3:c.570C>T  p.Ala190Ala | synonymous | 0.275 | n/a | n/a | n/a | -0.1 | n/a |
|  | rs5216 | 11: 17,408,838 | NM_000525.3:c.801C>G  p.Leu267Leu | synonymous | 0.017 | n/a | n/a | n/a | 2.21 | n/a |
|  | rs1800467 | 11: 17,408,831 | NM_000525.3:c.808C>G  p.Leu270Val | missense | 0.022 | 0.98  Deleterious | 0  Benign | 0.999656 Deleterious | 4.79 | 0.687162  Disease-causing |
|  | rs5215 | 11: 17,408,630 | NM_000525.3:c.1009G>A  p.Val337Ile | missense | 0.280 | 0.76  Tolerated | 0.002  Benign | 0.947433 Neutral | -2 | 1.00x10^-5^  Polymorphism-automatic |
|  | rs8175351 | 11: 17,408,496 | NM_000525.3:c.1143G>A  p.Lys381Lys | synonymous | 0.006 | n/a | n/a | n/a | 4.26 | n/a |
| ***BAD*** | rs34882006 | 11: 64,051,823 | NM_004322.3:c.18G>A  p. = *(p.Glu6Glu)* | synonymous | 0.0133 | n/a | n/a | n/a | 4.79 | n/a |
|  | rs2286615 | 11: 64,039,175 | NM_004322.3:c.288C>A  p.= *(p.Arg96Arg)* | synonymous | 0.0934 | n/a | n/a | n/a | 3.87 | n/a |

***** according to Human Genome Variation Society recommendations for the description of DNA sequence variants - v2.0 http://www.hgvs.org/mutnomen/recs-DNA.html

SIFT Pathogenicity score from dbNSFP (obtained from wANNOVAR): tolerated < 0.95, deleterious > 0.95

PolyPhen2 Pathogenicity score from dbNSFP (obtained from wANNOVAR): probably damaging > 0.85, possibly damaging 0.85-0.15, benign < 0.15

GERP++ Nucleotide conservation score from dbNSFP GERP (obtained from wANNOVAR): higher number is more conserved, > 0 is generally conserved

LRT Pathogenicity probability score from dbNSFP (obtained from wANNOVAR): closer to 1 is more likely to be damaging. Deleterious fulfils the following criteria: (i) from a codon defined by LRT as significantly constrained (original LRT score <0.001 and <1), (ii) the AA position has more than 10 eutherian mammal alignments. Neutral: the alternative AA is presented in at least one of the eutherian mammals, or the codon is not defined by LRT as significantly constrained; otherwise unknown.

MutationTaster Pathogenicity probability score from dbNSFP (obtained from wANNOVAR): closer to 1 is more likely to be damaging

Supplementary Table 3: Response to KDT in individuals with variants in *KCNJ11* and *BAD* with MAF<0.01 in this cohort

| **Gene** | **Variant nomenclature**  **(cDNA level and protein level)* or rs number (dbSNP)** | **Location build** | **Function class** | **SIFT pathogenicity score** | **Conserved?**** | **Mutation Taster classification** | **Number of cases with variant (genotype)** | **Description of diet response** |
| --- | --- | --- | --- | --- | --- | --- | --- | --- |
| ***KCNJ11*** | NM_00525.3:c.[1116C>T];[=]  p.= *(p.Ser372Ser)* | 11:17408523 | synonymous | - | - | - | 1  (CT) | Case 1: No change in seizures. Weaned off diet after 6 months |
|  | NM_00525.3:c.[912C>T];[=]  p.= *(p.Tyr304Tyr)* | 11:17408727 | synonymous | - | - | - | 1  (CT) | Case 2: No change in seizures. Weaned off diet after 3 months |
|  | NM_00525.3:c.[451G>A];[=]  p.Val151Met | 11:17409188 | missense | Deleterious (score 0.00) | Weakly conserved nucleotide; highly conserved amino acid | Disease-causing  (p-value 0.991) | 1  (GA) | Case 3: No change in seizures. Weaned off diet after 3 months |
|  | rs5214 | 11:17408550 | synonymous | - | GERP++ score -10.4 | - | 1  (GA) | Case 4: Partial responder in first 3 months, then improved response in second 3 months (>75% seizure reduction). Currently 6 months on diet. |
|  | NM_00525.3:c.[291C>T];[=]  p.= *(p.His97His)* | 11:17409348 | synonymous | n/a | n/a | n/a | 1  (CT) | Case 5: Around 40% reduction in seizures, and much more alert and interactive. Currently on diet for 3 months |
|  | rs112070496 | 11:17409531 | synonymous | - | GERP++ score 3.66 | - | 1  (AG) | Case 6: Partial responder in first 3 months, then improved response in second 3 months (>75% seizure reduction). Currently 6 months on diet. (same case as with rs5214) |
|  | NM_00525.3:c.[817A>G];[=]  p.Ser273Gly | 11:17408822 | missense | Deleterious (score 0.03) | Not conserved nucleotide; Highly conserved amino acid | Polymorphism  (p-value 1.0) | 1  (AG) | Case 7: Partial responder at the start, and more alert, interactive and vocal. Slight reduction in seizures did not last, so weaned off diet after 6 months |
|  | rs41282930 | 11:17408485 | missense | Deleterious (score 0.00) | GERP++ score 4.52 | Disease-causing. Score=0.630056 | 6  (all GC) | Case 8: According to seizure diary, seizures increased by 386% in first 3 months, compared to baseline. Parents describe that the diet had had no effect on seizure frequency, but the patient had increased awareness  Case 9: >50% seizure reduction in first 3 months. Seizure frequency increased during following 3 months and the patient was weaned off diet. Seizure frequency and severity further increased. Patient went back on diet but no improvement.  Case 10: Achieved >50% seizure reduction with KDT  Case 11: Achieved >90% seizure reduction with KDT  Case 12: Achieved >50% seizure reduction with KDT  Case 13: Achieved >50% seizure reduction with KDT |
| **BAD** | NM_004322.3:c.[142G>A];[=]  p.Ala48Thr*** | 11:64051699 | missense | Tolerated (score 0.68, median 4.32) | Not conserved nucleotide; moderately conserved amino acid | Polymorphism  (p-value 0.997) | 1  (GA) | Case 14: No change in seizures (possible increase). Weaned off diet after 3 months |
|  | rs75052600 | 11:64051853 | 5’ UTR | unknown | unknown | unknown | 1  (CT) | Case 15: No change in seizures, tired and irritable. Weaned off diet before 3-month point |
|  | NM_004322.3:c.-41G>A;[=]  p.?*** | 11:64051881 | 5'UTR | unknown | unknown | unknown | 1  (GA) | Case 16: Responder (50-75% seizure reduction). Weaned off diet after 6 months due to abdominal pain but went back on diet – initial good response |
|  | rs2286616 | 11:64039136 | synonymous | - | GERP++ score -2.44 | - | 2  (Both CT) | Case 17: Extreme responder – almost seizure-free. Seizure frequency variable after 3 years on diet  Case 18: Slight improvement in seizures but weaned off diet after 3 months due to compliance difficulties |
|  | NM_004322.3:c.[456G>A];[=]  p.= *(p.Gln152Gln)**** | 11:64037732 | synonymous | - | - | - | 1  (GA) | Case 19: Responder (50-75% seizure reduction). Remains on diet long-term. |
|  | rs59283629 | 11:64037679 | 3’ UTR | unknown | unknown | unknown | 3  (All CT) | Case 20: Responder (>75% seizure reduction). One year on diet  Case 21: No change in seizures. Currently at the 3-month point and weaning off diet  Case 22: No change in seizures, but much more alert. Currently on diet for 6 months |
|  | NM_004322.3:c. [226T>A];[=]  p.Tyr76Asn*** | 11:64039237 | missense | Deleterious (score 0.00, median 4.32) | Weakly conserved nucleotide; highly conserved amino acid | Polymorphism  (p-value 0.537) | 2  (Both TA) | Case 23: Seizure-free. Currently 6 months on diet  Case 24: No change in seizures. Off diet after 3 months |

*According to Human Genome Variation Society recommendations for the description of DNA sequence variants - v2.0 http://www.hgvs.org/mutnomen/recs-DNA.html

** Predictions of conservation for variants without a dbSNP ID are provided by Alamut, based on PhyloP score (nucleotide conservation) and cross-species alignment (amino acid conservation); GERP++ Nucleotide conservation scores from wANNOVAR are provided for variants with a dbSNP ID (higher number is more conserved, >0 is generally conserved).

***Variants not found in dbSNP (<http://www.ncbi.nlm.nih.gov/projects/SNP/>) or ExAC Browser (<http://exac.broadinstitute.org/>)

SIFT Pathogenicity scores (the closer the number is to 0, the more damaging the variant) are taken from Alamut reports for variants without a dbSNP ID and from wANNOVAR for variants with a dbSNP ID.

Mutation Taster classifications and p-values (an indication of the probability of the prediction; a value close to 1 indicates a high 'security' of the prediction) are taken from Alamut reports for variants without a dbSNP ID. Mutation Taster scores and classifications are taken from wANNOVAR for vairnats with a dbSNP ID: p-values not provided in wANNOVAR
